# Investigating urea is an ideal additive to the Accu-OptiClearing delipidation cocktail

**DOI:** 10.1038/s41598-025-18231-3

**Published:** 2025-12-14

**Authors:** Michael Siu-Lun Lai, Wai Man Chick, Rachel Man Hoi Law, Raymond Chuen-Chung Chang

**Affiliations:** 1 Laboratory of Neurodegenerative Diseases, School of Biomedical Science, LKS Faculty of Medicine, The University of Hong Kong, Pokfulam, Hong Kong SAR, China; 2 School of Biomedical Science, Faculty of Medicine Building, The University of Hong Kong, Room L4-49, Laboratory Block, 21 Sassoon Road, Pokfulam, Hong Kong, China

**Keywords:** Tissue clearing, Transparent brain, Myelin preservation, Tissue size preservation, Neural tracing, Three-dimensional imaging, Fluorescence imaging, Optical imaging

## Abstract

The brain is a complicated tissue with high lipid content. Different tissue clearing methods have been used to study its complex structures and functions. However, the balance between myelin preservation and clearing efficiency remains an obstacle in brain tissue clearing. In the previous study, Accu-OptiClearing has been developed to overcome this challenge by combining detergent and RI matching solution in the delipidation solution. This successfully preserves brain tissue structure and lipid content, but the clearing efficiency can be further improved. Here, we investigated the effects of adding urea as a non-detergent permeabilizing agent to the delipidation solution of Accu-OptiClearing by measuring the clearing efficiency, protein loss, tissue size change and myelin preservation. Different concentrations of urea (2 M, 4 M, 6 M and 8 M) were added to the 10% HxD-OPTIClear solution for comparison. From the results, we found that the 2 M urea additive can achieve sufficient improvement in clearing efficiency with unchanged tissue preservation and probe compatibility. These findings demonstrate that the addition of non-detergent urea could potentially modify the Accu-OptiClearing delipidation solution. Hence, it opens room for screening other non-detergent chemicals in optimizing the Accu-OptiClearing delipidation cocktail for future neuroscience studies.

## Introduction

As the most delicate organ in the body, the brain comprises over billions of neurons connected in different manners, forming a complex network^1^. Although 2D brain sections were commonly used in neuroscience studies, they are not sufficient to represent the integrity of the complicated connections inside the brain because of the information loss and tissue structural deformation during serial sectioning^2–4^. After the invention of confocal microscopes, optical sectioning has replaced physical sectioning by filtering the out-of-focus light during detection^5^. The technique has been further developed as the light sheet microscopes with minimized phototoxicity and increased imaging speed^6^. The optical sectioning technique serves as the basis for 3D brain imaging. However, the opacity of the brain tissues remains the major challenge. In recent years, the tissue clearing technique has been developed to minimize opaqueness by matching the refractive index (RI) within the tissues^7,8^.

The light intensity passing through an object determines the transparency, while the tissue clearing technique maximizes this light transmittance by minimizing light absorption and light scattering inside the tissues^9–11^. The hemoglobin in the blood is a common chromophore that absorbs light and limits the transparency of the brains^12,13^. Therefore, transcardial perfusion with buffered solutions is a common step in tissue clearing to wash out the blood and remove hemoglobin^11^. As a significant factor of opacity, light scattering results from the refraction and Fresnel reflection caused by the RI mismatches between different compartments inside the tissues^12^. Different tissue clearing methods correct the RI mismatches through different processes, including decolorization, delipidation, dehydration, hyperhydration and RI matching^8,14^. Based on the clearing processes and chemicals, the tissue clearing methods can be divided into three groups, including the hydrophobic methods, the hydrophilic methods and the hydrogel-based methods^8^. The hydrophobic methods, such as uDISCO (ultimate DISCO), FDISCO (DISCO with superior fluorescence-preserving capability) and SHANEL (small-micelle-mediated human organ efficient clearing and labeling), use organic chemicals to allow high clearing efficiency with the drawbacks of tissue shrinkage, chemical toxicity and incompatibility with specific probes^15–17^. The hydrophilic methods, such as Sca*l*e, Sca*l*eS, CUBIC (clear, unobstructed brain imaging cocktails and computational analysis) and CUBIC-X, use aqueous solutions and show high compatibility to different fluorescent probes but with tissue expansion from hydration^18–21^. The hydrogel-based methods, such as CLARITY (Clear Lipid-exchanged Acrylamide-hybridized Rigid Imaging/Immunostaining/In situ hybridization-compatible Tissue-hYdrogel), PACT (passive CLARITY technique) and SHIELD (stabilization under harsh conditions via intramolecular epoxide linkages to prevent degradation), allow efficient delipidation under hydrogel protection but with transient tissue expansion and high complexity of gel-setup procedures^22–24^. As a result, preservations of tissue size and lipid content are still two challenges of the conventional clearing methods.

To overcome these problems, we have previously developed Accu-OptiClearing (Accurate delipidation with Optimal Clearing). It is a strategy of combining the RI matching solution, Optical Properties-adjusting Tissue Clearing agent (OPTIClear), with the detergent to form delipidation solutions. It was found that the Accu-OptiClearing strategy is compatible with different detergents and has great effects on tissue size and lipid preservation by preventing excessive delipidation^25^. However, the clearing efficiency in large tissues remains a limitation in Accu-OptiClearing. Hence, we proposed that adding other non-detergent materials to the delipidation solution could enhance clearing efficiency while maintaining the lipid-preserving ability of the Accu-OptiClearing method.

Among different non-detergent materials, urea is selected because of its multiple functional properties and general usage in different tissue clearing methods. The small urea molecule contains both hydrogen donor and hydrogen acceptor groups^10^. This allows urea to form hydrogen bonds with water molecules and proteins, making it a hydrating agent and a denaturant, respectively^26^. The denaturant property of urea can disrupt the hydrogen bonds in the proteins and relax the protein scaffolds, including intercellular collagens^10^. This enhances the permeability of the tissue for the entry of clearing materials. On the other hand, the hydrating property of urea was manipulated to enhance the osmotic pressure inside the tissue to promote the detergent influx during clearing^11^. Based on these properties, urea was used in CUBIC as a component of the delipidation solution, in Sca*l*e as the hyperhydration agent, and in SUT (Scheme Update on tissue Transparency) clearing method^18–21,27^. By these denaturant and permeabilizing properties, urea was used as a potential additive to the delipidation solution in Accu-OptiClearing to enhance the clearing efficiency^28^. In this study, the clearing efficiency, tissue size preservation, tissue protein loss and probe compatibility of the urea additives have been assessed.

## Results

### Modification of Accu-OptiClearing delipidation solution by urea additives

**Fig. 1:**
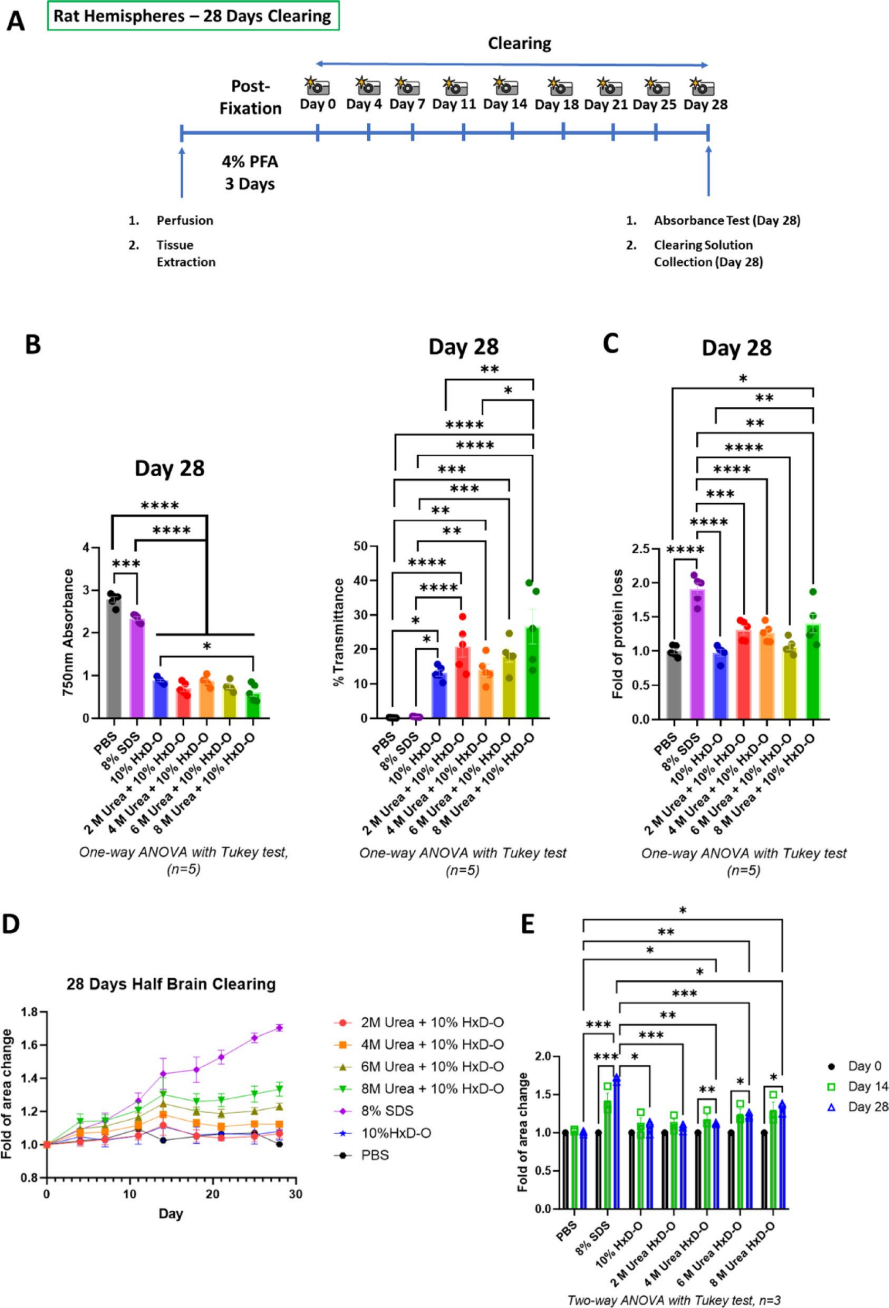
Tissue clearing of rat brain hemispheres with different concentrations of urea additives. (**A**) Workflow of the rat brain hemisphere clearing to assess clearing efficiency, tissue size change and protein loss. (**B**) Measured absorbance at 750 nm (left panel) and calculated % transmittance (right panel) on Day 28 after delipidation (*n* = 5). (**C**) Fold of tissue protein loss on Day 28 after delipidation (*n* = 5). The levels of protein loss after clearing (Day 28) were measured and normalized to the corresponding delipidation solutions before clearing (Day 0). Statistical analysis was performed using one-way ANOVA with Tukey’s multiple comparison test, *p ≤ 0.05, **p ≤ 0.01, ***p ≤ 0.001, ****p ≤ 0.0001. (**D**) Size changes of rat brain hemispheres in different clearing solutions with time (*n* = 3). (**E**) Fold of tissue area change on Day 0, Day 14, and Day 28 of delipidation. The tissue sizes at these time points were measured and normalized to the sizes of the corresponding tissues before clearing (Day 0) (*n* = 3). Statistical analysis was performed using two-way ANOVA with Tukey’s multiple comparison test, *p ≤ 0.05, **p ≤ 0.01, ***p ≤ 0.001, ****p ≤ 0.0001. All Data are presented as mean ± SEM.

In the previous study, 1,2-hexanediol (HxD) was found to be compatible with OPTIClear to form the 10% HxD-OPTIClear solution, which has the highest clearing efficiency in Accu-OptiClearing for rat brain hemispheres^25^. Therefore, 10% HxD-OPTIClear was used as the basal delipidation solution to test the effects of urea additives in this study. Different clearing solution candidates were prepared by adding serial concentrations of urea (2 M, 4 M, 6 M, and 8 M) to the 10% HxD-OPTIClear solution. These solution candidates, together with the 10% HxD-OPTIClear basal solution and the 8% sodium dodecyl sulphate (SDS) solution used in PACT^24^, were compared based on clearing efficiency, tissue protein loss, and tissue size change in clearing rat brain hemispheres. Phosphate-buffered saline (PBS) was used as the incubation solution for the uncleared controls. After post-fixation, the hemispheres were incubated in different solutions for 28 days. The tissue transparency and the tissue protein loss were assessed by measuring the absorbance of the tissues and the protein amounts in the clearing solutions on Day 28, respectively. During the clearing, bright-field images of the hemispheres were taken at 3–4 days intervals to monitor the tissue size change (Fig. 1A).

For the transparency examination, absorbance was measured at 750 nm, which is a wavelength in the infrared region to prevent interference from the yellow color of the cleared samples in Accu-OptiClearing. From the measured tissue absorbance at 750 nm, the transmittance percentage was calculated through the equation: % Transmittance = 10^−(Absorbance −2)^. Therefore, higher tissue transparency correlates to lower tissue absorbance and a higher transmittance percentage. From the results of calculated transmittance, the 10% HxD-OPTIClear solution and all the urea additive candidates achieved significantly higher transparency than the 8% SDS treated and the uncleared brain samples. Among different urea additive candidates, the 8 M urea + 10% HxD-OPTIClear solution showed the highest clearing efficiency, significantly higher than the 10% HxD-OPTIClear basal solution. In comparison, the 2 M urea + 10% HxD-OPTIClear solution showed the second-highest clearing efficiency (Fig. 1B).

The protein loss was assessed by measuring protein amounts in the clearing solutions after 28 days of incubation, in which a higher protein amount in each solution correlates to higher protein loss during delipidation in that condition. The 8% SDS solution was found to have the most protein loss, which is significantly higher than all other solutions. Among the clearing solution candidates, only the 8 M urea + 10% HxD-OPTIClear solution reported a significant increase in protein loss compared to the 10% HxD-OPTIClear basal solution and the PBS control. Other solutions showed no significant protein loss after clearing (Fig. 1C).

For the size change during the 28 days of clearing, the 8% SDS solution caused severe hemisphere swelling throughout the process. Among the clearing solution candidates, the concentrations of the urea additives were found to be positively correlated with the swelling effects of the tissues during clearing. Only the 2 M urea + 10% HxD-OPTIClear solution represented a similar tissue size change as the 10% HxD-OPTIClear condition and the uncleared PBS control during the 28-day clearing (Fig. 1D). The results were confirmed in the comparison of the tissue sizes on Day 0, Day 14 and Day 28. The 2 M urea + 10% HxD-OPTIClear solution was the only urea additive candidate that showed no significant change in tissue size after clearing (Fig. 1E).

**Fig. 2:**
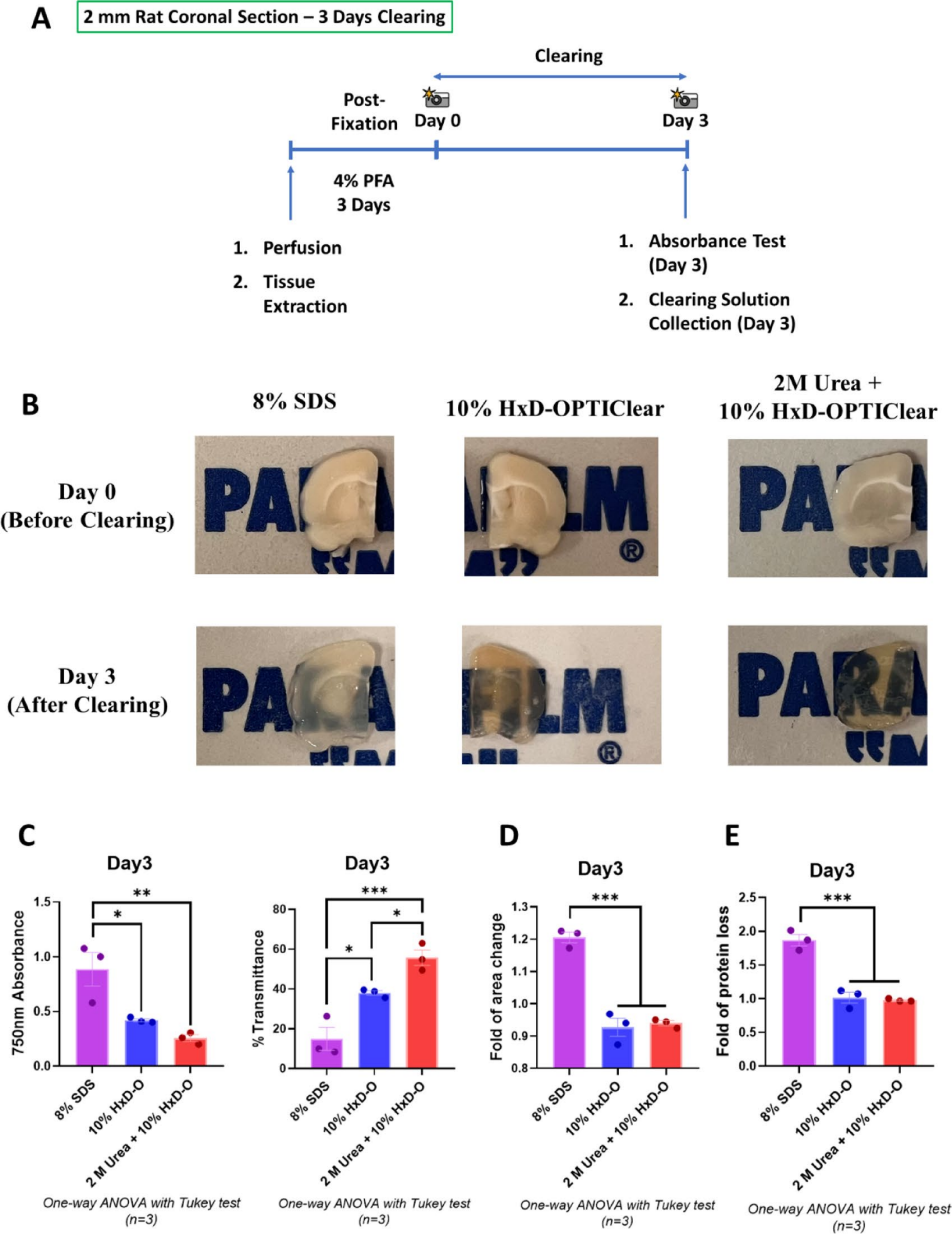
Addition of 2 M urea modifies delipidation solution in clearing 2 mm coronal rat brain sections. (**A**) Workflow of the 2 mm coronal rat brain section clearing to assess clearing efficiency, tissue size change and protein loss. (**B**) Representative images of the 2 mm coronal rat brain sections before and after clearing. (**C**) Measured absorbance at 750 nm (left panel) and calculated % transmittance (right panel) on Day 3 after delipidation. (**D**) Fold of tissue area change on Day 3 after delipidation. The tissue sizes after clearing (Day 3) were measured and normalized to the sizes of the corresponding tissues before clearing (Day 0). (**E**) Fold of tissue protein loss on Day 3 after delipidation. The levels of protein loss after clearing (Day 3) were measured and normalized to the corresponding delipidation solutions before clearing (Day 0). All Data are presented as mean ± SEM (*n* = 3). Statistical analysis was performed using one-way ANOVA with Tukey’s multiple comparison test, *p ≤ 0.05, **p ≤ 0.01, ***p ≤ 0.001, ****p ≤ 0.0001.

After screening the candidates with different urea concentrations in rat brain hemisphere clearing, the 2 M urea + 10% HxD-OPTIClear solution was selected as the optimized clearing solution based on its properties of enhanced clearing efficiency with preserved protein content and tissue size during delipidation. Hence, the effects of the 2 M urea additive to the clearing solution were further validated in smaller 2 mm rat brain sections after 3 days of clearing, in which the tissue absorbance, tissue size and tissue protein loss were measured (Fig. 2A). The 2 M urea + 10% HxD-OPTIClear condition was compared with the 10% HxD-OPTIClear basal solution and the 8% SDS solution (Fig. 2B). For the tissue absorbance and calculated transmittance, the brain sections cleared with the 2 M urea + 10% HxD-OPTIClear solution showed significantly higher transparency than the other two conditions on Day 3 (Fig. 2C). For the tissue size and protein loss, both 2 M urea + 10% HxD-OPTIClear and 10% HxD-OPTIClear clearing conditions showed no significant difference (Fig. 2D-E). Collectively, the addition of 2 M urea in the 10% HxD-OPTIClear solution enhances clearing efficiency while preserving the tissue size and protein content after the delipidation process in clearing small and large brain tissues.

### The modified Accu-OptiClearing is compatible with antibody and myelin staining

**Fig. 3:**
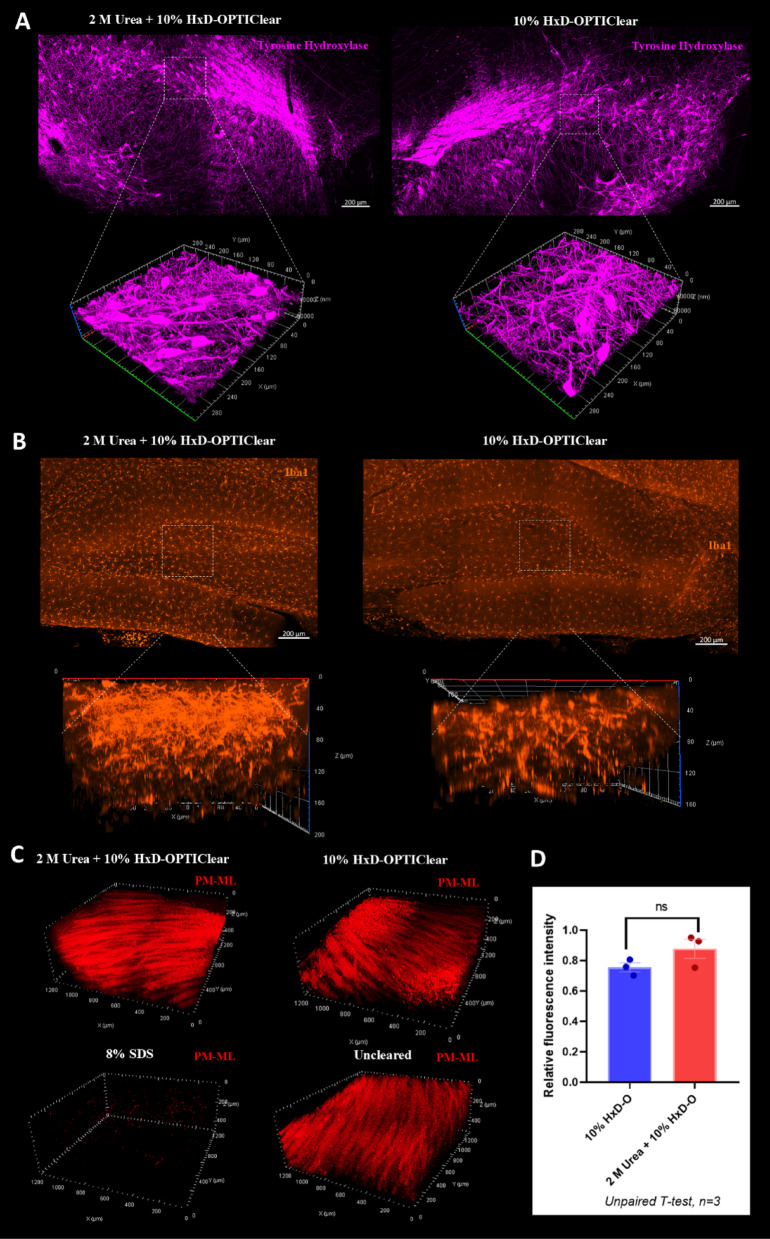
Assessment of probes compatibility with the 2 M urea + 10% HxD-OPTIClear solution. (**A**) Tyrosine hydroxylase (TH) staining of the 2 mm sagittal rat brain sections cleared with 2 M urea + 10% HxD-OPTIClear (left panel) and 10% HxD-OPTIClear (right panel). The upper panel is the orthogonal projection of the SN (10x), while the lower panel is the magnified z-stack image (20x). (**B**) Iba-1 staining of the 2 mm sagittal rat brain sections cleared with 2 M urea + 10% HxD-OPTIClear (z-depth = 200.79 μm) (left panel) and 10% HxD-OPTIClear (z-depth = 163.53 μm) (right panel). The upper panel is the orthogonal projection of the DG (10x), while the lower panel is the magnified z-stack image (20x). (**C**) Z-stack images (10x) of PM-ML staining in the mouse striatum cleared with 2 M urea + 10% HxD-OPTIClear, 10% HxD-OPTIClear and 8% SDS. SN, substantia nigra; DG, dentate gyrus. (**D**) Quantification of PM-ML staining fluorescence intensity in 2 M urea + 10% HxD-OPTIClear and 10% HxD-OPTIClear conditions relative to uncleared sample. All Data are presented as mean ± SEM (*n* = 3). Statistical analysis was performed using unpaired T-test, *p ≤ 0.05, **p ≤ 0.01, ***p ≤ 0.001, ****p ≤ 0.0001.

Next, the staining compatibility of using the 2 M urea + 10% HxD-OPTIClear solution in Accu-OptiClearing was tested by immunostaining and myelin staining. The staining results of this modified clearing solution were compared with those of the 10% HxD-OPTIClear basal solution. For the immunostaining, the antibodies of tyrosine hydroxylase (TH) and Iba-1 were used to stain the rat substantia nigra (SN) and hippocampus regions, respectively. In the TH staining, the stained dopaminergic neurons were clearly observed in both conditions (Fig. 3A). In the Iba-1 staining, the rat hippocampus cleared with the 2 M urea + 10% HxD-OPTIClear solution showed a higher z-depth (z-depth = 200.79 μm) than that cleared with the 10% HxD-OPTIClear solution (z-depth = 163.53 μm) (Fig. 3B). In summary, the addition of 2 M urea in 10% HxD-OPTIClear does not compromise the immunostaining signal quality but slightly enhances penetration of the antibodies.

For the myelin staining, PM-ML is a newly developed probe with aggregation-induced emission (AIE) properties. It allows strong near-infrared emission in the aggregation state after binding to the myelin sheath^29^. The PM-ML probe was used to stain the striatal regions of the sagittal mouse brain sections before overnight clearing by the 2 M urea in 10% HxD-OPTIClear, 10% HxD-OPTIClear and 8% SDS solutions. The 8% SDS solution removed almost all the myelin contents, and only weak PM-ML signals were detected. Conversely, both the 2 M urea + 10% HxD-OPTIClear and the 10% HxD-OPTIClear solutions preserved the lipid-rich myelin after clearing, and they showed a clear PM-ML staining as the uncleared sample without delipidation. (Fig. 3C). By quantifying the relative fluorescence intensity of the PM-ML staining signals, there is no significant difference between the original 10% HxD-OPTIClear solution and the newly optimized solution with 2 M urea additive (Fig. 3D). Collectively, the 2 M urea + 10% HxD-OPTIClear solution preserves the antigenicity and the myelin of the brain tissues during delipidation.

### Application of the modified Accu-OptiClearing in neural tracing of VTA-CA1 circuit

**Fig. 4:**
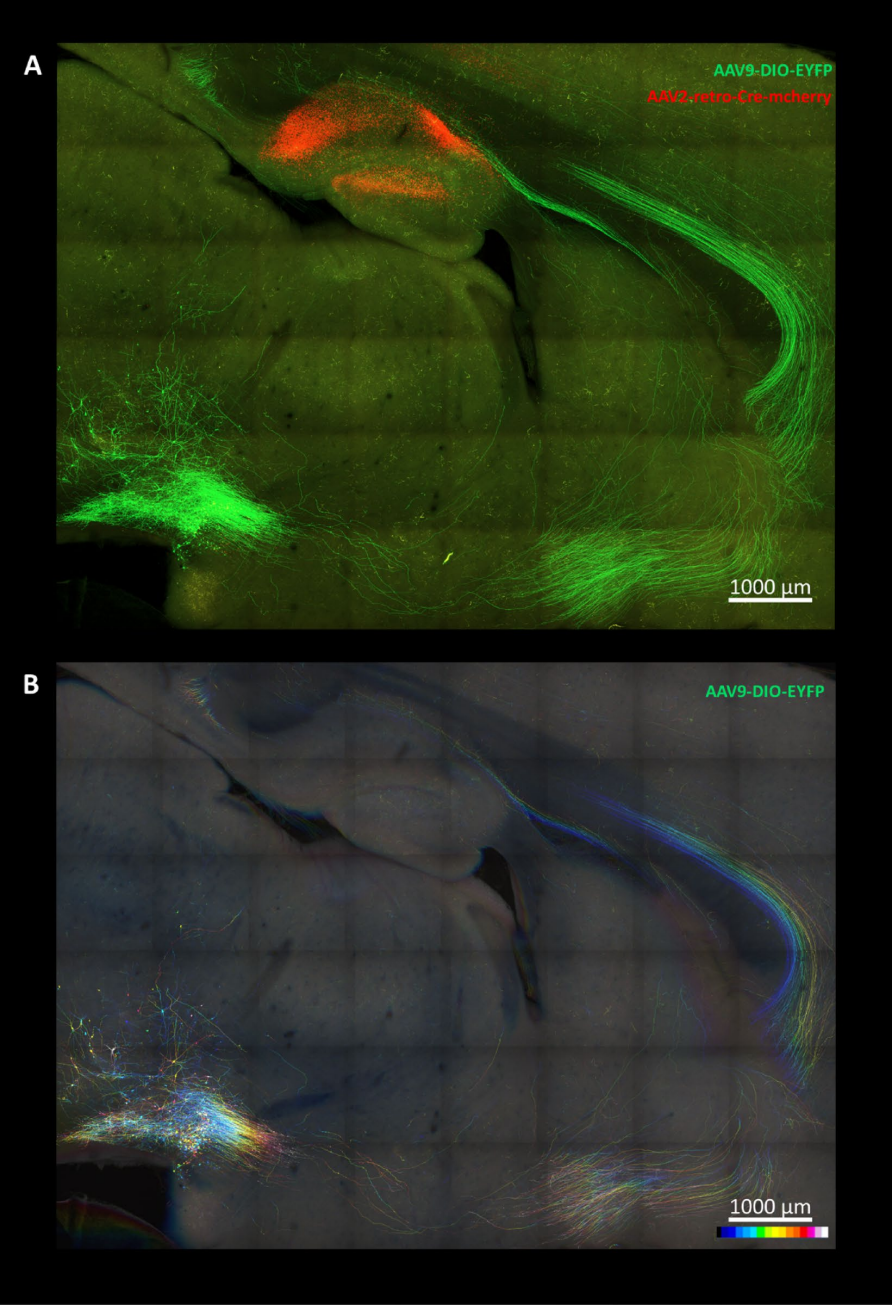
Application of the modified clearing solution in neural tracing. (**A**) Orthogonal projection of the AAV9-DIO-EYFP and the AAV2-retro-Cre-mCherry signals in the Antero-System, labeling the VTA-CA1 neural circuit (z-depth: 347.65 μm). (**B**) Color-coded projection of the anterograde AAV9-DIO-EYFP signals connecting VTA to CA1 in the same brain section (z-depth: 347.65 μm). VTA, ventral tegmental area; CA1, Cornu Ammonis 1.

Apart from the staining probes, endogenous fluorescence is another source of labeling different cells in the brain. Indeed, endogenous fluorescence transduced by viral vectors is commonly used to label neurons and their connections in neural tracing, a technique used to map neural circuits in neuroscience studies^30^. Hence, the compatibility of the 2 M urea + 10% HxD-OPTIClear solution with the virus-transduced endogenous fluorescence was assessed by tracing the known neural circuit connecting the ventral tegmental area (VTA) to the CA1. By using the Antero-System, a dual adeno-associated virus (AAV) tracing system designed in our previous study^31^, the VTA-CA1 circuit was specifically labeled by endogenous EYFP fluorescence. The signals were visualized by confocal microscope after clearing with the 2 M urea + 10% HxD-OPTIClear solution. The EYFP-labeled neurons in the VTA and their axonal projections towards CA1 were detected in a 3D manner, as shown in the orthogonal and color-coded projections (Fig. 4A-B). This shows that the 2 M urea + 10% HxD-OPTIClear solution did not damage the endogenous fluorescent signals in neural tracing.

## Discussion

Delipidation is a crucial process in minimizing the RI mismatches within the tissues during clearing, and it is a standard treatment in most of the hydrophilic, hydrophobic and hydrogel-based clearing methods^8^. This delipidation process removes lipid molecules and potentially causes tissue expansion through hydration^32,33^. Therefore, the potential lipid-rich structure disruption and tissue size change problems become common obstacles in maintaining tissue structural integrity during the development of clearing methods. In the previous study, our group has developed a strategy named Accu-OptiClearing to combine detergent with RI matching solution in the delipidation solution^25^. This allows the control of the delipidation extent and prevents excessive lipid removal. Although the tissue structural integrity can be maintained by preserving the lipid content and tissue size, the clearing efficiency of this partial delipidation needs to be improved in clearing rat brain hemispheres^25^. Here, we tried to modify the 10% HxD-OPTIClear delipidation solution in Accu-OptiClearing by enhancing the clearing efficiency while maintaining the tissue preservation ability. Urea was selected as the non-detergent additive in this study because of its hydrating and denaturing properties, which allow it to increase the fluidity and permeability of the cell membranes^28,34^. Then, we hypothesized that urea could facilitate the influx of the Accu-OptiClearing delipidation solution into the cells, resulting in a potentially enhanced clearing efficiency. From the transmittance measurement, the 8 M urea additive was found to have the highest transparency, which is significantly higher than the 10% HxD-OPTIClear basal solution in clearing half brains. Futhermore, the 2 M urea additive was found to have the second highest transparency in clearing hemispheres and significantly higher transparency than the 10% HxD-OPTIClear basal solution in clearing 2 mm brain sections. Hence, it shows that the addition of urea can improve clearing efficiency in both large and small brain tissues.

Apart from the potential expansion in delipidation, other processes in different clearing methods also challenge tissue size preservation. Hyperhydration in CUBIC-based clearing methods leads to tissue expansion^20,21^, while dehydration in the DISCO-based clearing methods leads to tissue shrinkage^15,17,35^. Recently, an innovative hydrophobic clearing method named SOLID (Suppressing tissue distortion based on synchronized dehydration/delipidation treatment with 1,2-hexanediol [1,2-HxD] mixtures) was developed using HxD in the gradient dehydration process^32^. HxD is a neutral delipidation agent used in the CUBIC-based clearing methods and Accu-OptiClearing to show strong lipid removal ability at low concentrations^25,36^. Conversely, it was found that HxD also has a strong dehydrating ability as an organic solvent at high concentrations. SOLID manipulates these properties of HxD and preserves tissue size after gradient dehydration/delipidation of increasing concentrations of HxD^32^. Besides, other clearing methods also reported unchanged tissue size after the treatment, including MACS (MXDA-based Aqueous Clearing System) and SHIELD^23,37^. In MACS, transient expansion of the tissues was caused by hyperhydration to enhance permeability, and the tissues were shrunk back to their original size after the treatment of the solution with high osmolality^37^. In SHIELD, the tissues were also transiently expanded after delipidation by SDS, but they were shrunk back to normal size after incubation in the RI matching solution^23^. Although SOLID, MACS and SHIELD can successfully preserve tissue size after the clearing procedure, the tissues were transiently expanded during clearing before they shrunk back to their original sizes^32,37,38^. This expansion-shrinkage process may induce potential distortions in the tissues. In this study, we tested the tissue size preservation of the Accu-OptiClearing delipidation solution after adding different concentrations of urea. As a hydrating agent, urea was reported to cause tissue expansion in the CUBIC-based and Sca*l*e-based clearing methods^18–21^. This is consistent with our results from the brain hemisphere clearing at high urea concentrations. The tissue expansion of the half brains is positively correlated with the concentrations of the urea additives. Among different urea additives, only the 2 M urea additive showed no significant swelling compared to the original tissue size. More importantly, the tissue sizes remain constant without transient expansion throughout the 28-day clearing process under the treatment of this 2 M urea additive. The tissue size preserving ability of the 2 M urea + 10% HxD-OPTIClear solution was validated in clearing 2 mm brain sections, in which no significant tissue size changes have been observed. As a result, we found that the OPTIClear in the clearing solution can preserve the tissue size at low urea concentration.

In addition to the tissue size, the protein content is another essential factor in tissue structural preservation. Some tissue clearing methods can potentially induce protein loss during the clearing process because of the denaturants, such as SDS and urea. For example, CLARITY and Sca*l*e clearing methods have been reported to induce ~ 8% and ~ 41% of protein loss after clearing, respectively^22^. Since the structural proteins in the extracellular matrix are essential in maintaining tissue structure, high-level protein loss may induce tissue distortion and deformation^39^. OPTIClear in the Accu-OptiClearing delipidation solution was reported to preserve the proteins in the presence of SDS denaturant^25^. Hence, we tested whether OPTIClear can preserve the protein content in different urea additives. Similar to the size change measurement, the OPTIClear cannot preserve the tissue protein level at high urea concentrations. The 8 M urea additive showed significantly higher protein loss than the 10% HxD-OPTIClear basal solution. Conversely, the 2 M urea additive showed no significant protein loss in clearing both half brains and 2 mm brain sections. Collectively, the 2 M urea + 10% HxD-OPTIClear solution was selected as the most optimized clearing solution in this study because of the balance between its enhanced clearing efficiency and unchanged tissue preservation ability.

The tissue protein content is not only related to the structural integrity but also crucial in preserving antigens and endogenously expressed proteins. This preservation was investigated by the immunostaining and neural tracing in this study, Immunostaining results showed that the addition of 2 M urea did not compromise the signal quality of the TH and Iba1 antibodies but also slightly improved the penetration of the Iba1 antibody. Moreover, the neural tracing of the VTA-CA1 circuit also demonstrated the compatibility of this modified clearing solution with the endogenous fluorescence transduced by viral vectors. Apart from the protein probes, lipophilic probes are also important to label myelin structures in brain studies. The lipid-preserving ability of Accu-OptiClearing was already confirmed by the DiI staining^25^. From the results of the PM-ML staining, it was found that the addition of 2 M urea into the 10% HxD-OPTIClear did not sacrifice the myelin preservation during delipidation. Altogether, the optimized 2 M urea + 10% HxD-OPTIClear solution is compatible with different labeling tools, including immunostaining, myelin staining and neural tracing.

Immunostaining, neural tracing and myelin staining are important labeling tools to study different neural structures in physiological and pathological conditions. Under physiological conditions, neural tracing and immunostaining were used to elucidate neuronal connections and corresponding cell types inside the brain, facilitating the understanding of the brain functions in connectomincs^40,41^. Under disease conditions, neural tracing and myelin staining could be used to monitor the propagation of pathological molecules and demyelination in neurodegenerative diseases^42–45^. Indeed, the application of Accu-OptiClearing with neural tracing and immunostaining was demonstrated in our previous study. This combination of techniques identified the 3D neural circuit with its postsynaptic neuronal subtype and monitored axonal degeneration in disease models^31^. Hence, the modified Accu-OptiClearing in this study can be potentially applied to broad neuroscience studies by allowing a 3D visualization of different neural structures.

By different tests, urea was shown to be compatible as an additive to the delipidation solution in Accu-OptiClearing. As a well-known reagent that is already widely used in different tissue clearing methods, the working concentration of urea is always 4 M in other clearing protocols. For instance, CUBIC and SUT used 25% urea (~ 4 M urea), while Sca*l*e used 4 M urea during clearing^18–21,27^. Surprisingly, the clearing efficiency of the 2 M urea additive was found to be higher than that of the 4 M urea additive for clearing rat hemispheres in this study. This may be due to the urea properties, in which the diffusion speed of the solution decreases with the concentration of urea^46–48^. Hence, the higher compatibility of 2 M urea concentration with the Accu-OptiClearing solution results in a balance between the solution diffusion speed and osmotic influx. However, the underlying mechanism needs to be further studied in the future. Nonetheless, the low concentration of urea was found to moderately improve the clearing efficiency in Accu-OptiClearing and prevent tissue swelling caused by hyperhydration observed in CUBIC and Sca*l*e.

In the clearing solution, the OPTIClear component was reported to prevent tissue expansion and protein loss caused by SDS in the previous study^25^. Consistently, this preserving ability was proved to be applicable to the urea additive in this study. This opens room for different non-detergent agents to modify the clearing solution in Accu-OptiClearing. Although the urea additive showed enhanced clearing efficiency and penetration depth of antibodies, the clearing cocktail can be further optimized by screening other hydrating and decolorizing additives in the next step. For the hydrating agent, formamide is an uncharged hydrating agent with a stronger permeabilizing property than urea. For the decolorizing agent, Quadrol (N, N,N’,N’-tetrakis(2-hydroxypropyl)ethylenediamine) is an aminoalcohol, which removes the heme chromophore by releasing hemin in CUBIC-based clearing methods^21,49^. Indeed, both formamide and Quadrol were shown to be compatible with urea in F-CUBIC (adding formamide to CUBIC)^50^, making them potential additives to the 2 M urea + 10% HxD-OPTIClear clearing solution from this study. On the other hand, the m-xylylenediamine (MXDA) used in the MACS clearing method was also reported to cause tissue hyperhydration while decolorizing heme chromophore by releasing iron ions^37^. Hence, it can be another potential additive to the Accu-OptiClearing delipidation solution in future studies.

In conclusion, the addition of urea to the delipidation solution can improve the clearing efficiency and antibody penetration in Accu-OptiClearing. The 2 M urea was screened as the optimal concentration, in which the OPTIClear solution can counteract the potential protein loss and tissue expansion during clearing. As a result, 2 M urea + 10% HxD-OPTIClear was found to be the optimized clearing solution because of its (a) improved clearing ability, (b) tissue structural preservation, and (c) compatibility with different probes. This enables the future application of this optimized clearing solution in neuroscience studies through neural tracing and myelin labeling.

## Methods

### Animals

All animal handling procedures and surgery in this study were conducted according to the guidelines from the Department of Health, the Hong Kong SAR government, and the Committee on the Use of Live Animals in Teaching and Research (CULATR) in the Comparative Medicine Research (CCMR), The University of Hong Kong (CULATR number 4782-18 and 22–237) with accreditation from the Association for Assessment and Accreditation of Laboratory Animal Care International. Sprague-Dawley (SD) rats (300 g) and C57BL/6J mice (3-month-old) were ordered from the CCMR, The University of Hong Kong.

The SD rats were housed in the capacity of a maximum of 3 animals per cage, while the C57BL/6J mice were housed in the capacity of a maximum of 5 animals per cage. All the cages were kept at constant temperature (20 ± 2 °C) and humidity (50 ± 10%). Food and water were available *ad libitum*. A 12:12 h light-dark cycle was maintained during husbandry at the CCMR, The University of Hong Kong.

### Accu-OptiClearing solutions preparation

For the RI matching solution, OPTIClear was prepared as described in the previous study^25^. In brief, 20% (w/v) N-methylglucamine (Sigma-Aldrich #66930), 32% (w/v) Iohexol (Sigma-Aldrich #D2158) and 25% (v/v) 2,2’-thiodiethanol (TDE) (Sigma-Aldrich #88559) were mixed in Milli-Q water. The solution was then adjusted to pH 7–8.

For the delipidation solution, 10% HxD-OPTIClear was prepared by mixing 10% HxD (v/v) with OPTIClear. On top of the 10% HxD-OPTIClear, different amount of the urea powder was dissolved in the solution to create different clearing solution candidates with different concentrations (2 M, 4 M, 6 M and 8 M) of urea additives.

### Transcardial perfusion and tissue post-fixation

Intraperitoneal injection (IP) of overdosed sodium pentobarbital (Alfasan, Holland) (120 mg/kg body weight) was performed to euthanize the C57BL/6J mice and the SD rats. After the euthanization, the rib cage of each animal was cut open, and a needle connecting the pump was inserted into the heart. The animals were first perfused with 0.9% saline followed by 4% paraformaldehyde (PFA) solution for 10–30 min, depending on the body size. The brains were harvested and immersed in 4% PFA solution at 4 °C for 3 days of post-fixation.

### Accu-OptiClearing

After post-fixation, the animal brains were washed with 1x PBS three times for 10 min each (3 × 10 min). Then, a brain matrix (Zivic Instruments, USA) was used to cut the brain tissues into different thicknesses. The rat brain hemispheres and the 2 mm coronal rat brain sections were used to test the properties of different clearing solution candidates. The 2 mm sagittal rat brain sections were used for neural tracing and immunostaining. The 1 mm sagittal mouse brain sections were used for PM-ML staining.

During the delipidation step, brain sections were incubated in the clearing solutions at 37 °C for different durations, depending on their thicknesses. The 1 mm brain sections were cleared overnight, while the 2 mm brain sections were cleared for 3 days. Conversely, the rat brain hemispheres were incubated in the clearing solutions at 37 °C for 28 days, with the solution renewed twice a week. After the delipidation, the tissues were washed with 1x PBS three times for 10 min each (3 × 10 min) to remove the clearing solutions. The tissues were then subjected to immunostaining or RI matching.

During the RI matching, the tissues were immersed in the OPTIClear solution at 37 °C for 2 days. After the RI matching step, the tissues were mounted onto 35 mm glass bottom dishes (MatTek, USA) with OPTIClear solution. A 24 mm x 24 mm coverslip (Thermo Fischer Scientific, USA) was used to cover each tissue during mounting. The mounted tissues were imaged under the confocal microscopes.

### Measurement of tissue transparency

After certain time points of delipidation, the cleared rat brain hemispheres and 2 mm sections were trimmed into a round shape, containing the brain regions ventral to the corpus callosum, with the same diameter as the wells in a 96-well plate. The trimmed brain tissues were incubated with the corresponding clearing solutions in the wells of the 96-well plate, defined as the tissue wells. Other wells were filled with the clearing solutions for blanking, defined as the solution wells. All bubbles were removed in the wells before absorbance measurement. The absorbance of each well in the 96-well plate was measured at 750 nm using the LabSystems Multiskan microplate reader (LabX, Canada). The finalized absorbance of the cleared tissues was calculated by subtracting the absorbance of the tissue wells from the absorbance of their corresponding solution wells. The % transmittance of the tissue was then calculated from the finalized absorbance by the equation: % Transmittance = 10^−(Absorbance −2)^.

### Measurement of tissue size change

The tissues were assumed to swell equally in all directions after delipidation, and the tissue area changes were measured to determine the tissue size changes. For the 2 mm coronal brain sections, bright-field images were taken on Day 0 and Day 3. For the hemispheres, the bright-field images were taken on Day 0, Day 4, Day 7, Day 11, Day 14, Day 18, Day 21, Day 25 and Day 28. The ‘polygon selections’ function in Fiji (Image J; NIH) was used to measure the tissue areas. The fold of area change was calculated by dividing the brain tissue areas at different time points by their corresponding areas on Day 0.

### Measurement of tissue protein loss

After delipidation, the clearing solutions were collected at the end of each process. Freshly prepared clearing solutions were used as the controls. The clearing solutions (40 µL) were mixed with 6x Laemmli sample buffer (8 µL). The mixtures were heated at 99 °C for 10 min to denature the proteins and were loaded onto the 10% Mini-PROTEAN^®^ TGX stain-free acrylamide gels (BioRad, USA). Electrophoresis was performed at 120 V for around 1.5 h. After the electrophoresis, the stain-free gels were visualized by UV detection in the GelDoc Go Imaging System (BioRad, USA). The measured signal intensity of each loaded lane was analyzed using the Image Lab software (BioRad, USA). The signal intensity of each sample was normalized by their corresponding solution control to calculate the fold of tissue protein loss.

### Immunostaining

The brain tissues were first incubated in the blocking solution containing 0.6 M glycine (Sigma-Aldrich #G7126), 0.2% v/v Triton^®^ X-100 (Sigma-Aldrich #T8532) and 3% v/v goat serum (Vector Laboratories #S-1000) in 1x PBS, at 37 °C for overnight. After washing with 0.2% PBS-Tween^®^ 20 (3 × 10 min), the brain tissues were stained by either TH antibody (Sigma-Aldrich #AB-152) or Iba-1 antibody (Wako #019-19741) in the antibody diluent, which contains 0.2% v/v Tween^®^ 20 (Sigma-Aldrich #P7949) and 3% goat serum in 1x PBS, in a 1:100 dilution at 37 °C for at least 2 days. After the primary antibody staining, the brain tissues were washed with 0.2% PBS-Tween^®^ 20 (6 × 30 min). The brain tissues were then stained by the goat anti-rabbit secondary antibody conjugated with Alexa647 (Thermo Fischer Scientific #A-21245) in a 1:100 dilution with antibody diluent at 37 °C for at least 2 days. After the secondary antibody staining, the brain tissues were washed with 0.2% PBS-Tween^®^ 20 (6 × 30 min). They were then subjected to the RI matching step in Accu-OptiClearing.

### PM-ML staining

PM-ML is a newly developed aggregation-induced emission (AIE)-active probe of myelin sheath with a high signal-to-noise ratio and photostability. The staining process was performed as previously described^29^. In brief, the 3 mL PM-ML staining solution was prepared by mixing 6 µL of 5mM PM-ML stock solution with 300 µL dimethyl sulfoxide (DMSO) (10%) and 2694 µL 1x PBS, so that the final concentration of the PM-ML is 10 µM in the staining solution. Each 1 mm mouse brain section was immersed in the 3 mL PM-ML staining solution for overnight shaking with 15 revolutions per minute (RPM) at room temperature. The brain tissues were rinsed with 1x PBS after overnight PM-ML staining. The brain tissues were then subjected to delipidation and RI matching in Accu-OptiClearing.

### Neural tracing

The Antero-System was applied to trace the VTA-CA1 circuit in rats following the procedures as described in the previous study^31^. In brief, stereotaxic injection was performed under the anesthesia of ketamine (80 mg/kg) and xylazine (8 mg/kg). The AAV9-DIO-EYFP (BrainVTA, China; titer: ≥2 × 10^12 vg/mL) was injected into the VTA (5.0 mm posterior, 0.7 mm lateral and 7.0 mm ventral to bregma), while the AAV2-retro-Cre-mcherry (BrainVTA, China; titer: ≥2 × 10^12 vg/mL) was injected into the CA1 (3.6 mm posterior, 2.0 mm lateral and 2.1 mm ventral to bregma) by the Hamilton 600 Series microliter syringes with 12^o^ beveled needles (Hamilton Company, USA) at a rate of 0.25 µl/minute with total volume of 0.5 µl per injection site. The rats were euthanized 3 weeks after the AAV injection and the brains harvested were subjected to Accu-OptiClearing for delipidation and RI matching after the post-fixation.

### Confocal imaging

The confocal imaging was performed using the LSM-800 and LSM-900 inverted confocal microscopes (Carl Zeiss, Germany). The Plan-Apochromat 10x Ph1 M27 (NA 0.45) and Plan-Apochromat 20x Ph2 M27 (NA 0.8) objective lenses were used. The results of the PM-ML staining, immunostaining and neural tracing were imaged using the ‘Tile’ and the ‘Z-stack’ functions at 1024 × 1024 frame size in the Zen 2.3 Blue Edition software (Carl Zeiss, Germany). The color-coded and orthogonal projections were generated by the Zen 2.3 Blue Edition (Carl Zeiss, Germany).

### Quantification of PM-ML fluorescence intensity

Orthogonal projections were generated to maximize the PM-ML staining signals in different z-layers. The mean intensity was measured and quantified in Fiji (Image J; NIH) as the fluorescence intensity for each sample. The relative fluorescence intensity was calculated by dividing the mean fluorescence intensity from each sample by that from the uncleared control samples.

### Data processing and statistical analysis

The confocal images were visualized and processed through Zen 2.3 Blue Edition (Carl Zeiss, Germany) to generate orthogonal and color-coded projections. All statistical analyses were analyzed by the GraphPad Prism software version 10.0 (GraphPad Software, USA), using unpaired T-test, one-way and two-way analysis of variance (ANOVA). All data were presented in the form of mean ± SEM. p-value ≤ 0.05 was considered significant in the analysis.

## Data Availability

The original data in this study are available from the corresponding author upon request.
